# Specific functions of single pistil S-RNases in *S*-gene homozygous *Pyrus* germplasm

**DOI:** 10.1186/s12870-023-04605-0

**Published:** 2023-11-20

**Authors:** Yongjie Qi, Zhenghui Gao, Na Ma, Liqing Lu, Fanjun Ke, Shaoling Zhang, Yiliu Xu

**Affiliations:** 1 Key Laboratory of Horticultural Crop Germplasm Innovation and Utilization(Co-Construction By Ministry and Province), Institute of HorticultureAnhui Academy of Agricultural Sciences, Hefei, 230031 China; 2 College of Horticulture, Nanjing Agricultural University, Nanjing, 210095 China; 3 Anhui University of Chinese Medicine, Hefei, 230012 China

**Keywords:** *Pyrus*, *S*-gene homozygote, Single S-RNase, Proteomic analysis, Specific function

## Abstract

Gametophytic self-incompatibility (SI) is regulated by *S*-allele recognition; that is, pollen in a style with the same *S*-genotype will undergo programmed cell death and stop growing so that it is unable to complete double fertilization, ultimately resulting in the SI response. S-RNase is the female determinant of SI in pear (*Pyrus*). In the *Pyrus* genome, there are two different *S-RNase* alleles at the *S*-locus, which generate two different S-RNase products in the pistil. The extracted S-glycoprotein is actually a protein complex. In this study, artificial self-pollination was conducted at the bud stage to overcome SI in ‘Huanghua’ (*S*_*1*_*S*_*2*_) pear. Seven plants homozygous for *S*_*1*_*-RNase* and four homozygous for *S*_*2*_*-RNase* were selected from the selfed progeny of ‘Huanghua’ by *S*-gene molecular identification biotechnology. We investigated the function of single S-RNases isolated from the pistils of *S*-gene homozygous *Pyrus* germplasm. The pollen of ‘Huanghua’ could smoothly pass through the style of the *S*-gene homozygous germplasm and complete fertilization. S-RNases were extracted from flower styles of different genotypes and used to treat different types of pollen. The S-RNase from ‘Huanghua’ completely inhibited the growth of *S*_*1*_*S*_*2,*_* S*_*1*_*S*_*1*_, and *S*_*2*_*S*_*2*_ pollen, while the S-RNase from homozygous germplasm allowed some *S*_*1*_*S*_*2*_ pollen and different single genotypes of pollen to continue growing. These results further validate the core events of SI including cytoskeleton depolymerization and programmed cell death. By iTRAQ-based proteomic analysis of style proteins, a total of 13 S-RNase-related proteins were identified. In summary, we have created reliable *S-RNase* gene homozygous germplasm, which will play a crucial role in further research on SI in pear and in the development of the pear industry.

**Supplementary Information:**

The online version contains supplementary material available at 10.1186/s12870-023-04605-0.

## Introduction

Self-incompatibility (SI) in flowering plants is a genetic mechanism that prevents inbreeding and promotes outcrossing. In many species, SI is controlled by a single multi-allelic* S* locus [1]. In gametophytic self-incompatible (GSI) species of Rosaceae [2], Solanaceae [3], and Scrophulariaceae [4], the *S* locus encodes an allele-specific ribonuclease (S-RNase) that is expressed in the style. This S-RNase is responsible for inhibiting the growth of pollen tubes with the same *S* allele [5]. The pollen determinant is a single *S*-locus (haplotype) *F-box* gene (SLF/SFB) in *Prunus* species but is likely controlled by multiple *F-box* genes in some Rosaceae species and Solanaceae [6–9]. S-RNase can enter the pollen tube and degraded by SLF/SFB proteins associated with the ubiquitin-26S proteasome in non-self pollen tubes [10, 11]. According to the previous study, there are at least 6 methods that can be used for identified* S*-genotype [12–14]. The *S*-genotypes have been a useful reference for arranging suitable pollinators in Rosaceae species orchards and selecting hybrid parents in breeding.

Pear (*Pyrus*), in the family Rosaceae*,* has an S-RNase-based GSI system. In the past decade, a series explored the mechanism of GSI in pear. For example, the breakdown of SI in ‘Jinzhui’, ‘Yanzhuang’, ‘Zaoguan’, ‘Sha 01’ and ‘Xinxue’ were surveyed based on the inheritance of S-RNase alleles [15–19]. Using an in vitro system, we identified the characteristics of the S-RNase that specifically inhibits self-pollen germination and tube elongation [20–23]. Further studies showed that S-RNase induces the depolymerization of the actin cytoskeleton and DNA degradation in self-generated pollen tubes [24–26]. To further identify whether the results obtained in vitro reflect what happens in vivo, we evaluated the nuclear DNA of pollen tubes after different pollination events. In a previous study, the ABA concentration was increased at 24 h after pollination but decreased at 48 h [27]. Three ABRE-binding factors may be involved in GSI by regulating the expression of genes related to pollen tube growth [28]. Self S-RNase represses the expression of an upstream regulator (*PbABF.D.2*) of two *leucine-rich* repeat extension genes (*PbLRX.A2.1* and *PbLRX.A2.2*), causing a decrease in pollen tube growth [29].

Pomologists have focused on the S-RNase in the *Pyrus* style as the key protein controlling SI in pear. In the *Pyrus* genome, there are two different *S-RNase* genes at the same *S-RNase* alleles, which generate two different S-RNase products in the pistil. The extracted S-glycoprotein is actually a protein complex comprising these two products. However, because it is difficult to separate and purify each S-glycoprotein component individually, there are few reports on the functional characteristics of each single pistil S-RNase [30]. To overcome the above issues, we generated S-gene homozygous germplasm and then extracted and purified single S-RNase products. Through comparing the different characteristics of these single S-RNases expressed in the pistil, we determined how each single S-RNase regulates pollen recognition and pollen tube growth, and clarified the effects of homozygous pollen on the ability of each S-RNase to trigger programmed cell death (PCD) and alter the cytoskeleton. The methods used here provide new opportunities to explore the specific functions of single pistil S-RNases in *Pyrus*, which may be relevant for clarifying the molecular mechanisms that function in pear pistils and pollen. More significantly, this research demonstrates the use of a new system to study the mechanism of pear GSI by detailed analyses of *S-RNase* gene homozygous germplasm.

## Materials and methods

### Plant materials

Adult trees of the pear varieties ‘Huanghua’ (*Pyrus pyrifolia*, H–H, *S*_*1*_*S*_*2*_), ‘Xizilv’ (*Pyrus pyrifolia*, XZL,* S*_*1*_*S*_*4*_), and ‘Nijisseiki’ (*Pyrus pyrifolia*, NI, *S*_*2*_*S*_*4*_) growing in the orchards of Nanjing Agricultural University, Jiangsu, China were used in this study. The leaves, pistils, and pollen were collected and stored at -80°C for later analyses. *S-RNase* gene homozygotes were identified from the seedlings of the selfed progeny of ‘Huanghua’. The *S*_*1*_*-*homozygote H-7 (*S*_*1*_*S*_*1*_) and the *S*_*2*_*-*homozygote H-9 (*S*_*2*_*S*_*2*_) were used in this study.

### Bud stage artificial self-pollination

Artificial self-pollination of ‘Huanghua’ at the bud stage (4, 6, and 8 days before full bloom) was carried out in 2008. Before anthesis, two to three flowers per inflorescence were selected, emasculated, and enclosed in insect-proof bags to prevent contamination after artificial self-pollination. Fruit set was recorded 3 weeks later and fruits were collected when mature.

### PCR amplification and sequence analysis of genomic S-RNases

Genomic DNA was extracted from young leaves of each cultivar using the CTAB (cetyl trimethyl ammonium bromide) method (Macklin, Shanghai, China). PCR was performed with the consensus primers, forward primer (PF) (5-TTTACGCAGCA ATATCAGC-3) and reverse primer (PR) (5-AC(A/G)TTCGGCCAAATAATA-3) [31]. Amplifications with the following cycle parameters: 3 min at 94℃ for pre-denaturation, followed by 5 cycles of 30 s at 94℃, 30 s at 50℃, and 45 s at 70℃, then 30 cycles of 30 s at 94℃, 30 s at 48℃, and 45 s at 70℃, with a final extension for 7 min at 70℃. *S*_*1*_*-RNase* and *S*_*2*_*-RNase* alleles were discriminated on the basis of fragment length.

### S-gene quantitative real-time PCR assay

The *S*_*1*_*-RNase* allele-specific primers were *S*_*1*_-F and *S*_*1*_-R. The *S*_*2*_*-RNase* allele-specific primers were *S*_*2*_-F and *S*_*2*_-R (Supplemental Table 1). The transcript level of each gene in each sample was compared with that of *Actin* (AF386514). The RT-PCR system was as follows: 5μL 5 × PCR buffer, 4 μL MgCl_2_ (25 mmol L^−1^), 1.25 μL dNTP mixture (10 mmol L^−1^), 0.5 μL specific primers (10 μmol L^−1^), 1.25 μL 20 × Eva green fluorescent dye, 1.5 U Taq enzyme (2.5 U·μL^−1^), 1 μL template, and ddH_2_O to 25 μL. The sample was pre-degenerated at 95℃ for 5 min, 95℃ for 15 s, and 65℃ for 35 s over 40 cycles. A relative quantification method was used to calculate relative gene transcript levels [32].

### Analysis of nuclear DNA content by flow cytometry

Nuclear protoplasm was isolated by chopping 100 mg young leaf tissue with a sharp scalpel in a glass Petri dish containing 500 μL LB01 lysis buffer. The nuclei were stained with propidium iodide (PI) at 100 mg·mL^−1^ and incubated with 100 mg·mL^−1^ RNase for 15–30 min prior to analysis. After isolation, nuclei were fixed in ice-cold 3:1 fixative (ethanol/glacial acetic acid) and stored in 70% (v/v) ethanol at -20 °C. The PI-stained nuclei suspensions were analyzed with an Epics XL flow cytometer (Beckman Coulter, Miami, FL, USA) and sorter equipped with an argon ion laser at λ = 514 nm adjusted to a 100-mW power output [33]. Integral fluorescence and the height and width of fluorescence pulses emitted from the nuclei were collected through a 620 nm long-pass filter.

### Preparation, concentration, and activity of S-RNase

Four grams of styles were prepared for isolating each S-RNase following our previously described method [20, 22]. The isolated S-RNase sample was stored in Eppendorf tubes at -80℃. The S-RNase concentration and activity were determined by the methods of Bradford [34] and Brown and Ho [35], respectively.

### Pollination test and segregation of S-alleles

To check cross-(in) compatibility in the three cultivars/lines, self-pollination and cross-pollination tests were conducted in ‘Huanghua’, ‘H-7’, and ‘H-9’. Field pollination experiments were carried out in 2014. Pistils were harvested and fixed in FAA solution (5% v/v formalin, 5% v/v acetic acid, 45% v/v ethanol) 48 h after self-pollination or cross-pollination. After the epidermis was removed, the pistils were examined under a microscope equipped with a BH2-RFL-T2 ultraviolet light source and an Osram HBO 100 W/2 high-pressure mercury lamp (Olympus, Tokyo, Japan) [36]. To study the segregation of the *S-RNase* gene, seedling genotypes were detected by PCR using the primer set PF/PR for *S-RNase*.

### Pollen culture and depolymerization assays of filamentous actin in vivo

Pollen grains were pre-cultured for 2 h at 25℃ in a basal medium in the dark [20]. The basal medium consisted of a 2-(N-morpholine)-ethanesulfonic acid (MES)-NaOH buffer supplemented with 10% (w/v) sucrose, 15% (v/v) polyethylene glycol 4000, 0.01% (w/v) H_3_BO_3_, 0.07% (w/v) Ca(NO_3_)_2_ 4H_2_O, 0.02% (w/v) MgSO_4_·7H_2_O and 0.01% (w/v) KNO_3_, pH 6.0–6.5. After pre-culture, H-7 stylar S-RNase was added to the medium as an SI challenge, and H-9 stylar S-RNase was added to the medium as a compatible treatment. Medium without S-RNase was used as the control.

The final activity of the S-RNases in the basal medium was 0.15 U. An F-actin depolymerization assay was conducted and the amount of F-actin present in the samples was quantified as previously described [37]. A confocal microscope (LSM700, Zeiss, Jena, Germany) was used to examine the levels of actin depolymerization. Phalloidin fluorescence divided by ethidium bromide (EB) fluorescence was used as an index of actin filament levels. The bound phalloidin and EB were eluted with methanol and quantified by spectrofluorometry with excitation and emission wavelengths of 492 and 514 nm, respectively, for phalloidin, and 513 and 615 nm, respectively, for EB.

### Evaluation of programmed cell death in pollen tubes

We evaluated PCD in pollen tubes by terminal-deoxynucleotidyl transferase-mediated nick-end labeling (TUNEL) staining as previously described [30]. After fixing the samples in 4% (w/v) paraformaldehyde for 2 h, the pollen tubes were transferred to 70% (v/v) ethanol and incubated overnight at -20℃. The Dead End Fluorometric TUNEL system (Promega, Beijing, China) was used to examine PCD in the samples. The TUNEL signal was detected at excitation and emission wavelengths of 540 and 620 nm, respectively, and the DAPI signal was detected at excitation and emission wavelengths of 360 and 420 nm, respectively. Positive TUNEL staining in the pollen tubes was indicative of PCD.

### Protein extraction, iTRAQ labeling and strong cation exchange (SCX) fractionation

One gram of frozen style from H–H, *S*_*1*_*S*_*1*_, and *S*_*2*_*S*_*2*_ was ground to a powder in liquid nitrogen. Each sample included three biological replicates. The dry powder was dissolved in 0.6 mL lysis buffer (1 M sucrose, 0.5 M Tris 8.0, 0.1 M KCl, 50 mM ascorbic acid, 1% NP 40, 1% DOCNa, 10 mM EDTA, 10 mM DTT, 1% protease inhibitor mixture and 1% phosphatase inhibitor mixture) and placed on ice for 10 min [38]. The protein concentration was quantified using the Bio-Rad Protein Assay Kit (Bio-Rad, Hercules, CA, USA) following the manufacturer’s instructions.

From every sample, 100 μg protein was digested with Trypsin Gold (Promega, Madison, WI, USA) at a protein: trypsin ratio of 30:1 for 16 h at 37 °C. After trypsin digestion, the peptides were dried by vacuum centrifugation, and then dissolved in 0.5 M TEAB using 8-plex iTRAQ reagent [39]. The samples were marked with iTRAQ labels as follows: iT-S_1_S_2_, iT-S_1_S_1_, iT-S_2_S_2_. The peptides were labeled with isobaric tags at 25℃ with 2 h. The iTRAQ labeled peptide mixture was reconstituted in 2 mL buffer A (25 mM NaH_2_PO_4_ in 25% ACN, pH 2.7) and then loaded onto an Ultremex SCX column (particle size, 5 μm). The elution of products was detected by measuring absorbance at 214 nm. Finally, the eluted peptides collected in 20 fractions were desalted on a Strata X C18 column and then vacuum-dried [40].

Data were acquired from the Triple TOF 5600 System equipped with a pulled quartz tip as transmitter and a Nanospray III source, with the following settings: 30 psi curtain gas, 150℃ interface heater temperature, 15 psi atomization gas, and 2.5 kV ion spray voltage. A RP (≥ 30,000 FWHM) was used for operating the MS for TOF–MS scanning [41].

### iTRAQ protein identification and quantification

The raw data files obtained from the Triple TOF 5600 system were converted into MGF files using 5600 MS converter and then the MGF files were searched. For iTRAQ quantification, peptides for quantification were automatically selected by an algorithm to calculate reporter peak area, error factor (EF), and *p*-value (using the default parameters in the Mascot software package). Proteins showing 1.5-fold or higher difference in abundance between the treated and control samples and a *p-*value of less than 0.05 were identified as differentially expressed proteins (DEPs). Quantification was performed at the peptide level according to the procedure described at http://www.matrixscience.com/help/quant_statistics_help.html. Each treatment was repeated once for the iTRAQ analysis. The mass spectrometry proteomics data were stored in the PRIDE partner repository with the data set identifier PXD0043543 in the ProteomeXchange [42].

## Results

### Overcoming SI to obtain homozygous S-RNase gene germplasm

**Fig. 1:**
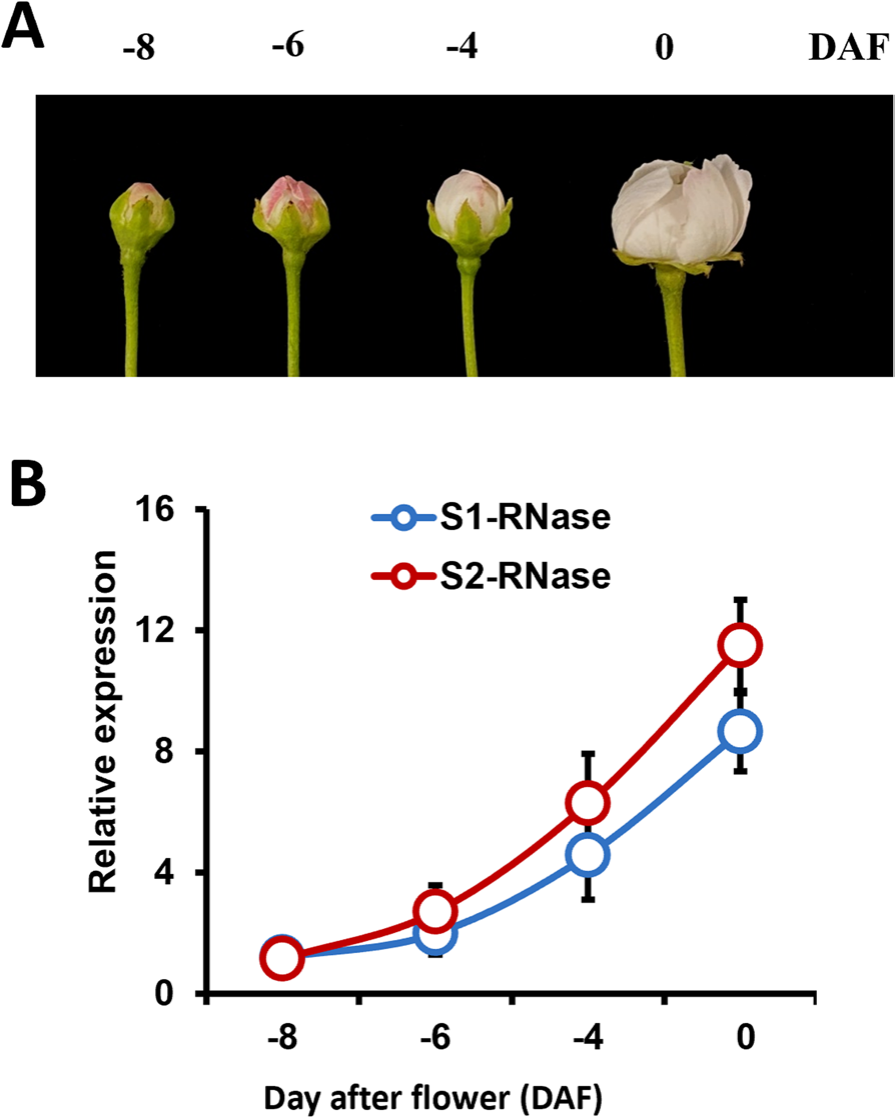
Spatio-temporal expression pattern of S-RNase in the floral tissues of ‘Huanghua’ pear at different developmental stages. **A** ‘Huanghua’ flower buds at different developmental stages [-8 days after flowering (DAF), -6 DAF, -4 DAF and 0 DAF]; **B** RT-PCR analysis of the relative transcript levels of S1-RNase and S2-RNase alleles in ‘Huanghua’

**Fig. 2:**
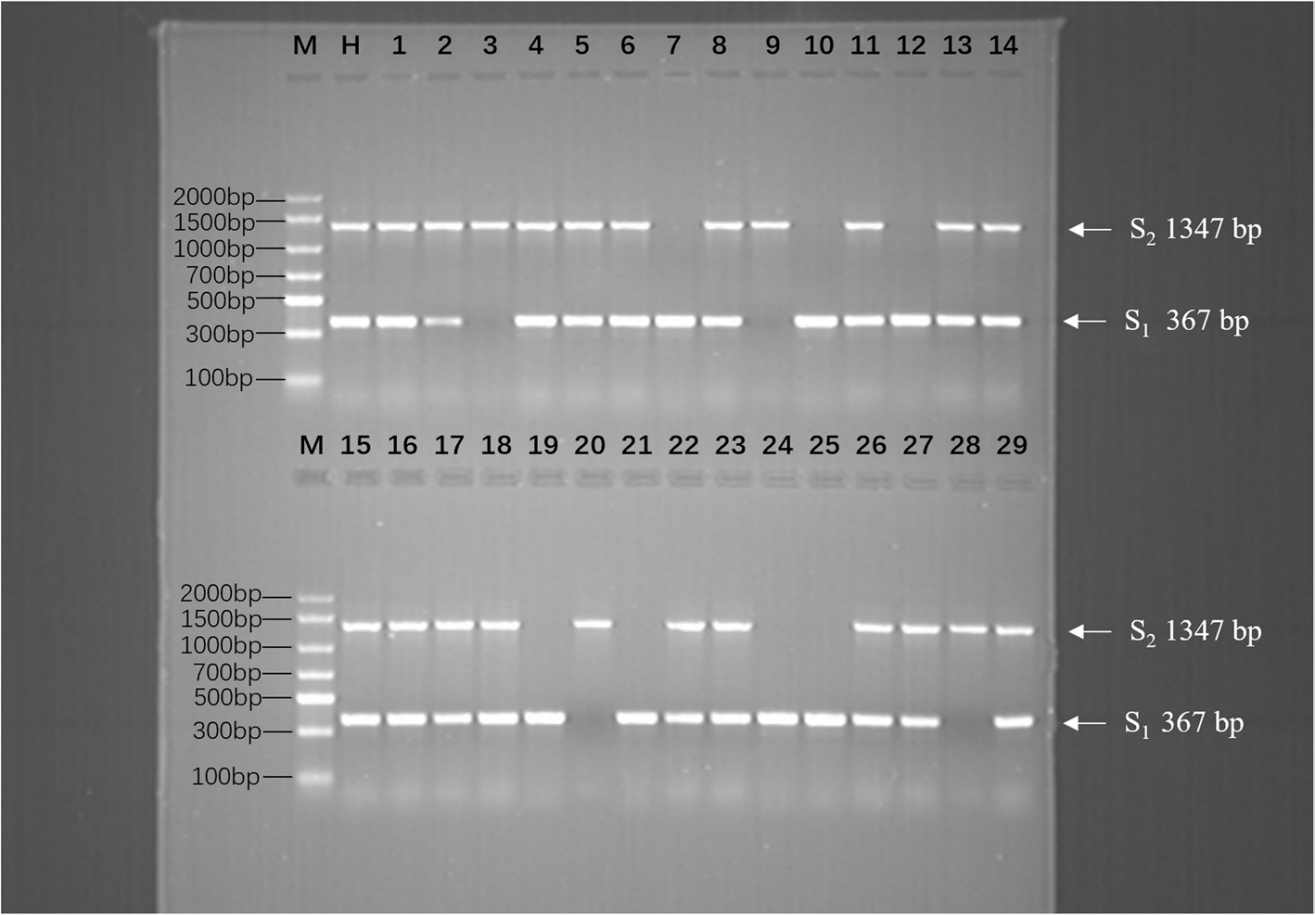
Identification of the S-genotype in ‘Huanghua’ and its selfed progeny at the bud stage. M. Marker; H. ‘Huanghua’; 1–29. ‘Huanghua’ selfed progeny

*Pyrus* is a typical gametophytic SI fruit tree. Analyses of the spatio-temporal expression patterns of *S*_*1*_*-RNase* and* S*_*2*_*-RNase* in the floral tissues of ‘Huanghua’ pear at different developmental stages [-8 days after flowering (DAF), -6 DAF, -4 DAF, and 0 DAF] revealed an increasing trend in their transcript levels until flowering (Fig. 1). Bud pollination was conducted to overcome SI in this cultivar and promote self-pollination. Fruit set and the average number of seeds after self-pollination were investigated after bud pollination (Table 1). Under natural conditions, ‘Huanghua’ showed the highest fruit set (43%) when it was self-pollinated at the bud stage at -6 DAF. When pollinated at the bud stage at 0 DAF, the fruit setting rate was the lowest, which was only 1%. In the spring of 2008, a total of 29 progenies were obtained from 74 seeds of ‘Huanghua’. To determine the *S*-genotypes of the selfed progeny of ‘Huanghua’, S-RNase alleles were amplified by PCR from the genomic DNA of all individual progeny. Genomic PCR using the primers PF and PR yielded a 367-bp fragment corresponding to the *S*_*1*_*-RNase* allele and a 1347-bp fragment corresponding to the *S*_*2*_*-RNase* allele from the selfed progeny of ‘Huanghua’ (Fig. 2).

**Table 1:** Comparison of fruit set and selfed progeny among different bud stage artificial self-pollination in ‘Huanghua’

Days before flowering	Number of pollinated flowers	Fruited flowers	Fruit set ratio (%)	Number of seeds	Number of self-pollinated progenies
8	100	16	16.0	26	6
6	100	43	43.0	45	23
4	100	9	9.0	3	0
0	100	1	1.0	0	0

‘Huanghua’ and its selfed progeny were analyzed to give an accurate estimation of nuclear DNA content. Controls containing 2content (2C) DNA showed peak 1 at the position (channel 100) that had been determined by analyzing standards prepared from known diploid ‘Huanghua’. The histograms of its selfed progeny with 2C DNA showed this same peak 1 at channel 100 (Supplemental Fig. 1). These results strongly indicated that all seedlings produced from the ‘Huanghua’ selfed progeny were diploids, thus, no spontaneous triploids were detected by flow cytometry analysis. It can be inferred that each individual ‘Huanghua’ selfed progeny contained two *S-RNase* alleles in its genomic DNA. Through the identification of the *S*-genotype and *S*-gene copy number, seven ‘Huanghua’ selfed progeny (No. 7, 10, 12, 19, 21, 24 and 25) were identified as *S*_*1*_*S*_*1*_ homozygotes, four selfed progeny (No. 3, 9, 20 and 28) were identified as *S*_*2*_*S*_*2*_ homozygotes, and the remaining 18 offspring were identified as *S*_*1*_*S*_*2*_ heterozygotes (Fig. 2). We performed χ^2^ tests on the segregation ratios observed in the ‘Huanghua’ selfed progeny, *S*_*1*_*S*_*1*_: *S*_*1*_*S*_*2*_: *S*_*2*_*S*_*2*_ = 7:18:4 (χ^2^ = 2.31<χ^2^_0.05,2_ = 5.99), it fits Mendelian segregation.

### Specific recognition functions between pollen and pistil in different homozygotes

We investigated the fruit set rate after self-pollination and cross-pollination with ‘H–H’ (Huanghua, *S*_*1*_*S*_*2*_), ‘H-7’ (*S*_*1*_*S*_*1*_ homozygote, no. 7 selfed progeny of Huanghua), and ‘H-9’ (*S*_*2*_*S*_*2*_ homozygote, no. 9 selfed progeny of Huanghua). ‘H–H’, ‘H-7’, and ‘H-9’ exhibited SI after self-pollination. Pollinations between various pairs of these three varieties were then conducted. When pollinated with ‘H–H’ (*S*_*1*_*S*_*2*_) pollen, 67% of ‘H-7’ (*S*_*1*_*S*_*1*_) flowers and 74% of ‘H-9’ (*S*_*2*_*S*_*2*_) flowers set fruit, displaying cross-compatibility; however, the reciprocal pollination resulted in only a few ‘H–H’ (*S*_*1*_*S*_*2*_) flowers setting fruit (Table 2). Cross-pollination between ‘H-7’ (*S*_*1*_*S*_*1*_) and ‘H-9’ (*S*_*2*_*S*_*2*_) displayed typical cross-compatibility.

**Table 2:** Fruit set percentage after cross- and self-pollinations of different *Pyrus* S-gene homozygote germplasm materials

Crosses for pollination	Number of pollinated flowers	Fruited flowers	Fruit set ratio (%)	Number of seeds per fruit	(In) Compatible
H–H(*S*_*1*_*S*_*2*_) selfed	100	0	0.0	0	SI
H-7(*S*_*1*_*S*_*1*_) selfed	100	1	1.0	0	SI
H-9 (*S*_*2*_*S*_*2*_) selfed	100	2	2.0	0	SI
H-7(*S*_*1*_*S*_*1*_) × H-H(*S*_*1*_*S*_*2*_)	100	67	67.0	4.5	CC
H-9 (*S*_*2*_*S*_*2*_) × H-H(*S*_*1*_*S*_*2*_)	100	74	74.0	4.8	CC
H–H(*S*_*1*_*S*_*2*_) × H-7(*S*_*1*_*S*_*1*_)	100	3	3.0	0	CI
H–H(*S*_*1*_*S*_*2*_) × H-9 (*S*_*2*_*S*_*2*_)	100	6	6.0	0	CI
H-7(*S*_*1*_*S*_*1*_) × H-9 (*S*_*2*_*S*_*2*_)	100	52	52.0	4.2	CC
H-9 (*S*_*2*_*S*_*2*_) × H-7(*S*_*1*_*S*_*1*_)	100	65	65.0	4.6	CC

**Fig. 3:**
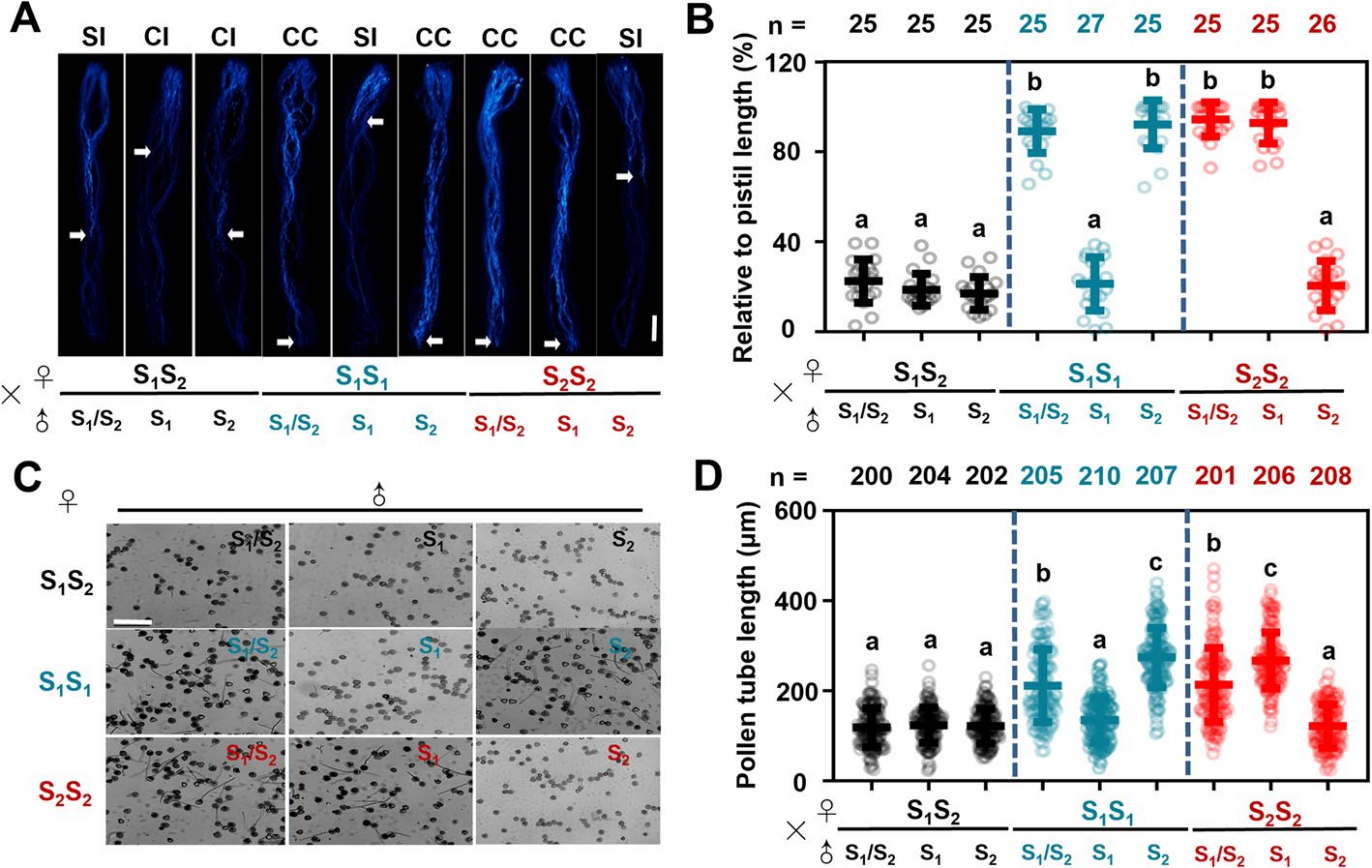
Pollen performance among self- and cross-pollinations in *S*-gene homozygote germplasm. **A** Micrographs showing pollen performance under self-pollination and cross-pollination in ‘*S*_*1*_*S*_*2*_’, ‘*S*_*1*_*S*_*1*_’ and ‘*S*_*2*_*S*_*2*_’. Arrow indicates the longest pollen tube in the pistil. Bar = 100 mm. **B** Pollen tube lengths relative to pistil length. Data are mean values and standard errors of three replicates. Different letters indicate significant differences as determined by ANOVA followed by Tukey’s multiple comparison test (*P* < 0.05). **C** Typical images of *S*-gene homozygote germplasm pollen tubes under in vitro culture conditions with different S-proteins (1.25 μg·μL^–1^). Bar = 100 μm. **D** Analyses of S-gene homozygote germplasm pollen tube length. At least 500 measurements were performed. Effect of S-proteins on pollen germination and growth of pollen tubes after 12 h of culture. Different letters indicate significant differences as determined by ANOVA followed by Tukey’s multiple comparison test (*P* < 0.05)

Pollen tube growth was subsequently investigated approximately 48 h after self-pollination or cross-pollination between various pairs of ‘*S*_*1*_*S*_*2*_’, ‘*S*_*1*_*S*_*1*_’, and ‘*S*_*2*_*S*_*2*_’ For all three types, after self-pollination, the pollen tubes could not complete normal development in the pistil or reach the ovary for fertilization. The ‘*S*_*1*_*S*_*2*_’ pollen was able to penetrate ‘*S*_*1*_*S*_*1*_’ or ‘*S*_*2*_*S*_*2*_’ pistils. However, ‘*S*_*1*_*S*_*1*_’ or ‘*S*_*2*_*S*_*2*_’ pollen could not penetrate ‘*S*_*1*_*S*_*2*_’ pistils (Fig. 3A, B). Notably, after cross-pollination between ‘*S*_*1*_*S*_*1*_’ and ‘*S*_*2*_*S*_*2*_’, the pollen tubes were able to reach the ovary. These results were consistent with the fruit set rate after self-pollination or cross-pollination in ‘*S*_*1*_*S*_*2*_’, ‘*S*_*1*_*S*_*1*_’, and ‘*S*_*2*_*S*_*2*_’.

Using the primers PF and PR, we amplified the genomic DNA of progeny of different cross-pollinations with ‘*S*_*1*_*S*_*1*_’ and ‘*S*_*2*_*S*_*2*_’ (Table 3). The bands *S*_*1*_ and *S*_*2*_ could be detected in all progeny of reciprocal cross-pollinations between ‘*S*_*1*_*S*_*1*_’ and ‘*S*_*2*_*S*_*2*_’. When ‘*S*_*1*_*S*_*1*_’ or ‘*S*_*2*_*S*_*2*_’ was the female parent and ‘*S*_*1*_*S*_*2*_’ was the male parent, the *S*-genotype of all cross-progeny was *S*_*1*_*S*_*2*_*.* When ‘XZL (*S*_*1*_*S*_*4*_)’ pollen was used to pollinate ‘*S*_*1*_*S*_*1*_’, the S-genotype of all cross-progeny was *S*_*1*_*S*_*4*_; however, the backcross showed typical cross-incompatibility. Similar results were also obtained in the reciprocal cross-pollination between ‘NI(*S*_*2*_*S*_*4*_)’ and ‘*S*_*2*_*S*_*2*_’. These results indicate that there is a specific recognition function between the pollen of the *S*-gene homozygotes and the pistil with the same *S*-locus.

**Table 3:** S-genotype in progeny obtained by different cross-pollinations of ‘H-7’ and ‘H-9’

Crosses for pollination	Number of pollinated flowers	Number of seeds obtained	Number of progeny genotyped	*S*-gene type
H-H(*S*_*1*_*S*_*2*_) × H-7(*S*_*1*_*S*_*1*_)	100	0	0	/
H-H(*S*_*1*_*S*_*2*_) × H-9 (*S*_*2*_*S*_*2*_)	100	0	0	/
H-7(*S*_*1*_*S*_*1*_) × H-9(*S*_*2*_*S*_*2*_)	100	218	169	*S*_*1*_*S*_*2*_
H-9(*S*_*2*_*S*_*2*_) × H-7(*S*_*1*_*S*_*1*_)	100	299	216	*S*_*1*_*S*_*2*_
H-7(*S*_*1*_*S*_*1*_) × H-H(*S*_*1*_*S*_*2*_)	100	301	246	*S*_*1*_*S*_*2*_
H-9(*S*_*2*_*S*_*2*_) × H-H(*S*_*1*_*S*_*2*_)	100	355	273	*S*_*1*_*S*_*2*_
H-7(*S*_*1*_*S*_*1*_) × XZL(*S*_*1*_*S*_*4*_)	100	167	129	*S*_*1*_*S*_*4*_
XZL(*S*_*1*_*S*_*4*_) × H-7(*S*_*1*_*S*_*1*_)	100	0	0	/
H-9(*S*_*2*_*S*_*2*_) × NI(*S*_*2*_*S*_*4*_)	100	182	136	*S*_*2*_*S*_*4*_
NI(*S*_*2*_*S*_*4*_) × H-9(*S*_*2*_*S*_*2*_)	100	0	0	/

### Effect of single S-RNase on homozygote pollen germination and pollen tube growth

The in vitro system of pear SI is relatively mature. Here, we used in vitro experiments to verify the SI responses of pear *S*-gene homozygotes. Three types of pollen, ‘*S*_*1*_/*S*_*2*_’ (H–H), ‘*S*_*1*_’ (H-7) and ‘*S*_*2*_’ (H-9), were cultured in liquid medium, to which S_1_-RNase and S_2_-RNase were then added as appropriate. Liquid medium containing ‘H–H’ stylar S-RNase (S_1_S_2_) was used as the control. As expected, ‘H–H’ stylar S-RNase inhibited the growth of all three types of pollen tubes (Fig. 3C). In S_1_-RNase liquid medium, pollen-tube elongation of ‘*S*_*1*_’ pollen was significantly inhibited with a mean pollen tube length of 115 ± 12.3 µm (*P* < 0.05, *n* = 210); while the ‘*S*_*1*_/*S*_*2*_’ and ‘*S*_*2*_’ pollen both grow normally, with mean pollen tube lengths of 210 ± 15.6 µm (*P* < 0.05, *n* = 205) and 280 ± 20.7 µm (*P* < 0.05, *n* = 207), respectively. In S_2_-RNase liquid medium, ‘*S*_*2*_*S*_*2*_’ pollen tubes were strongly inhibited, with a mean pollen tube length of 108 ± 10.1 µm (*P* < 0.05, *n* = 208); while the ‘*S*_*1*_/*S*_*2*_’ and ‘*S*_*1*_’ pollen tubes were not inhibited, with mean pollen tube lengths of 213 ± 12.1 µm (*P* < 0.05, *n* = 201) and 275 ± 18.9 µm (*P* < 0.05, *n* = 206), respectively (Fig. 3D). These results show that each single S-RNase has a significant inhibitory effect on the germination and growth of pollen containing the same *S*-gene type.

### Each single homozygous S-RNase induces alterations in the actin cytoskeleton and PCD in homologous pollen tubes in vitro

**Fig. 4:**
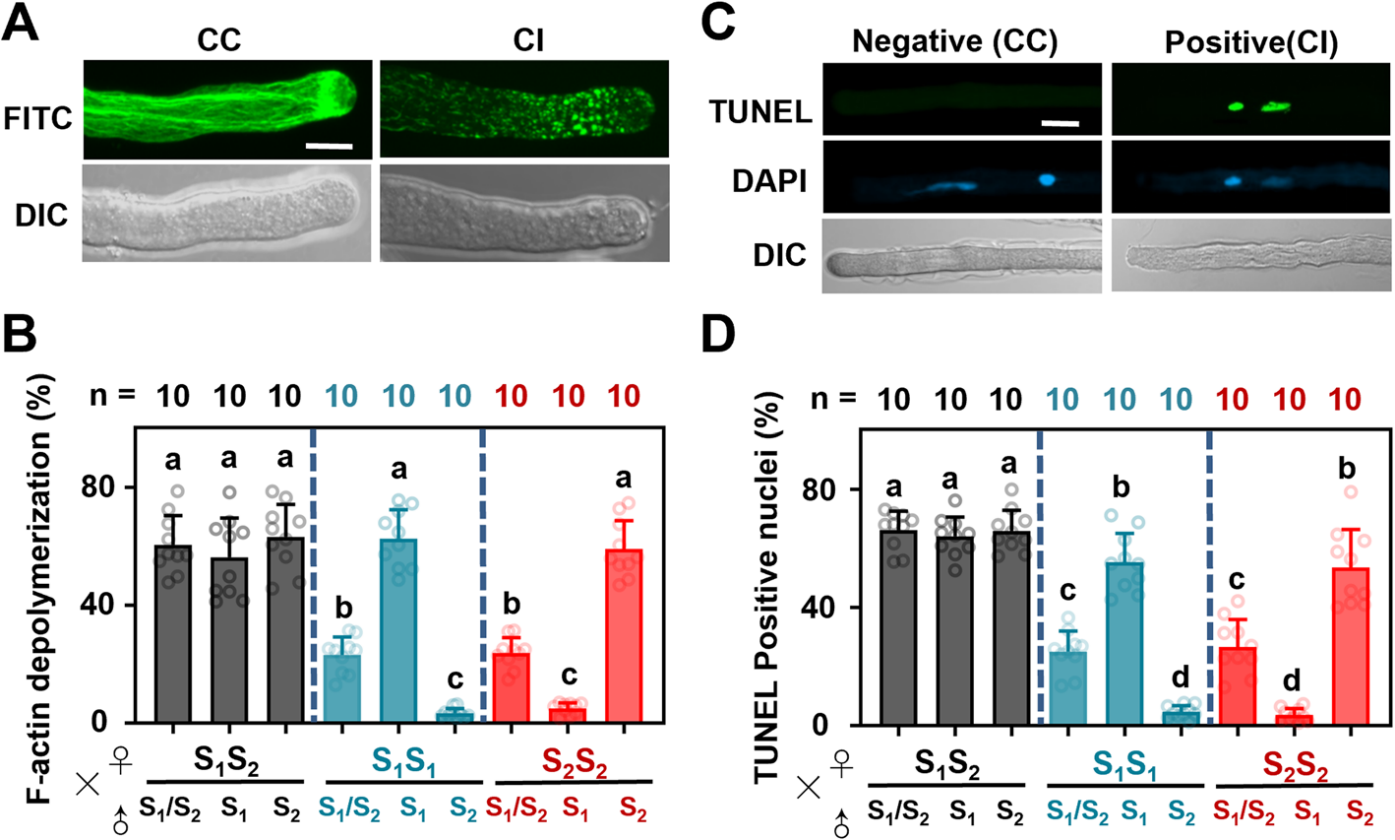
Each single homozygous S-RNase induces alterations in the actin cytoskeleton and DNA degradation in self-pollen and pollen tubes in vitro. **A** Typical images of actin cytoskeletal configuration in cross-incompatible and cross-incompatible pollen tubes after 30 min of various treatments. Bar = 20 mm. **B** Quantification of pollen tubes showing F-actin depolymerization in response to 30 min of various treatments. Different letters indicate significant differences, as determined by ANOVA followed by Tukey’s multiple comparison test (*P* < 0.05; n = 10); error bars indicate standard error. **C** TUNEL-based detection of programmed cell death (PCD) in pollen tubes. Typical images of TUNEL signals in pollen tubes during cross-compatible (CC, left) and cross-incompatible (CI, right) responses. DAPI staining results indicate that the TUNEL-CI signals are localized in the nuclear DNA. “Positive” indicates that PCD has occurred in the pollen tube, “negative” indicates no PCD. Bar = 10 μm. **D** Number of pollen undergoing PCD among cross and self-pollinations in ‘S1S2’, ‘S1S1’ and ‘S2S2’. Samples were processed for TUNEL staining, followed by statistical analysis. Experiment was repeated three times; each experiment included at least 100 measurements. Asterisk indicates significant statistical difference between CC treatment and CI as evaluated by ANOVA and Tukey’s test: *P* < 0.05; *n* = 10; error bars indicate standard error

The actin cytoskeleton of pollen tubes forms a dynamic cell framework that supports numerous fundamental cellular processes and is essential for their highly specialized polarized tip growth. Thus, we examined the effects of each single homozygous S-RNase on depolymerization of the actin cytoskeleton in homologous pollen tubes. We observed massive actin depolymerization in pear pollen tubes within minutes of an incompatible S-RNase challenge (Fig. 4A). We quantified the degree of F-actin depolymerization in response to 30 min of various treatments (Fig. 4B). The ‘H–H’ stylar S-RNase (S_1_S_2_) led to significant actin depolymerization in ‘*S*_*1*_/*S*_*2*_’, ‘*S*_*1*_’, and ‘*S*_*2*_’ pollen tubes. In S_1_-RNase liquid media, ‘*S*_*2*_’ pollen tubes grew normally, while F-action depolymerization was highly significant in ‘*S*_*1*_’ pollen tubes, 68.34% (*P* < 0.05; *n* = 10). Similar results were obtained in S_2_-RNase liquid media, in which the actin cytoskeleton remained intact in ‘*S*_*1*_’ pollen tubes, but showed a significant level of depolymerization in ‘*S*_*2*_’ pollen tubes, 67.25% (*P* < 0.05; *n* = 10). In S_1_-RNase and S_2_-RNase liquid media, the actin depolymerization rates in ‘*S*_*1*_*S*_*2*_’ pollen tubes were 19.21% and 21.32%, respectively (*P* < 0.05; *n* = 10). These findings show that each single homozygous S-RNase significantly depolymerized the actin cytoskeleton in homologous pollen tubes.

Senescent cells are precisely eliminated via PCD. Therefore, we used the TUNEL staining technique to assess DNA-strand breaks, which are indicative of PCD, in pear pollen tubes (Fig. 4C). The PCD ratio was the same, 58.43%, in ‘*S*_*1*_’ pollen tubes treated with S_1_-RNase (*P* < 0.05; *n* = 10) and ‘*S*_*2*_’ pollen tubes treated with S_2_-RNase (*P* < 0.05; *n* = 10) (Fig. 4D). These results show that the *S*-gene homozygotes generated in this study conformed to the characteristics of SI.

### iTRAQ-based proteomic analysis of style proteins and S-RNase-related proteins in Huanghua and its homozygous S-genotype offspring

**Fig. 5:**
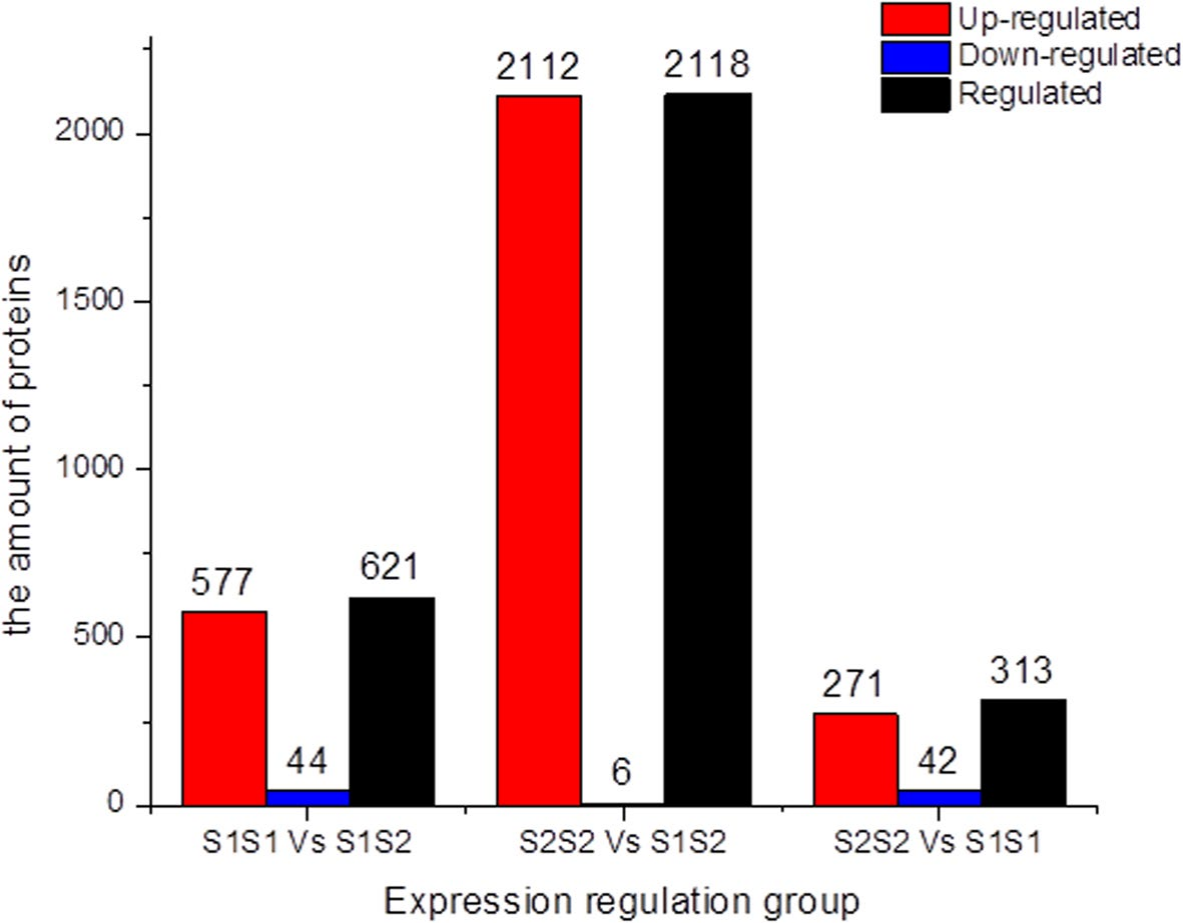
Bar chart showing the number of differentially expressed proteins (DEPs) among ‘S1S2’, ‘S1S1’, and ‘S2S2’

We used iTRAQ technology to investigate the protein expression characteristics of S-RNases in *S*-gene homozygous germplasm. By using MaxQuant to search the raw mass spectrometry data files against the protein database, a total of 30,503 identified spectra, 13,568 peptides, and 4,472 proteins were identified in the styles of Huanghua (*S*_*1*_*S*_*2*_) and its homozygous S-genotype offspring (*S*_*1*_*S*_*1*_ and *S*_*2*_*S*_*2*_). These identified proteins were predicted to be located in extracellular regions, cytoplasm, nucleus, mitochondria, Golgi apparatus, endoplasmic reticulum, peroxisomes, vacuoles, nucleoplasm, and other cellular compartments. The number of DEPs among the three varieties was calculated (Fig. 5). According to the overall distribution of these proteins, the highest number of DEPs was between *S*_*2*_*S*_*2*_ and *S*_*1*_*S*_*2*_, while the lowest number of DEPs was between *S*_*2*_*S*_*2*_ and *S*_*1*_*S*_*1*_. This indicated that the protein expression patterns of *S*_*2*_*S*_*2*_ and *S*_*1*_*S*_*1*_ were more similar to each other, while both offspring samples showed significant differences in protein expression when compared with the parent *S*_*1*_*S*_*2*_.

**Fig. 6:**
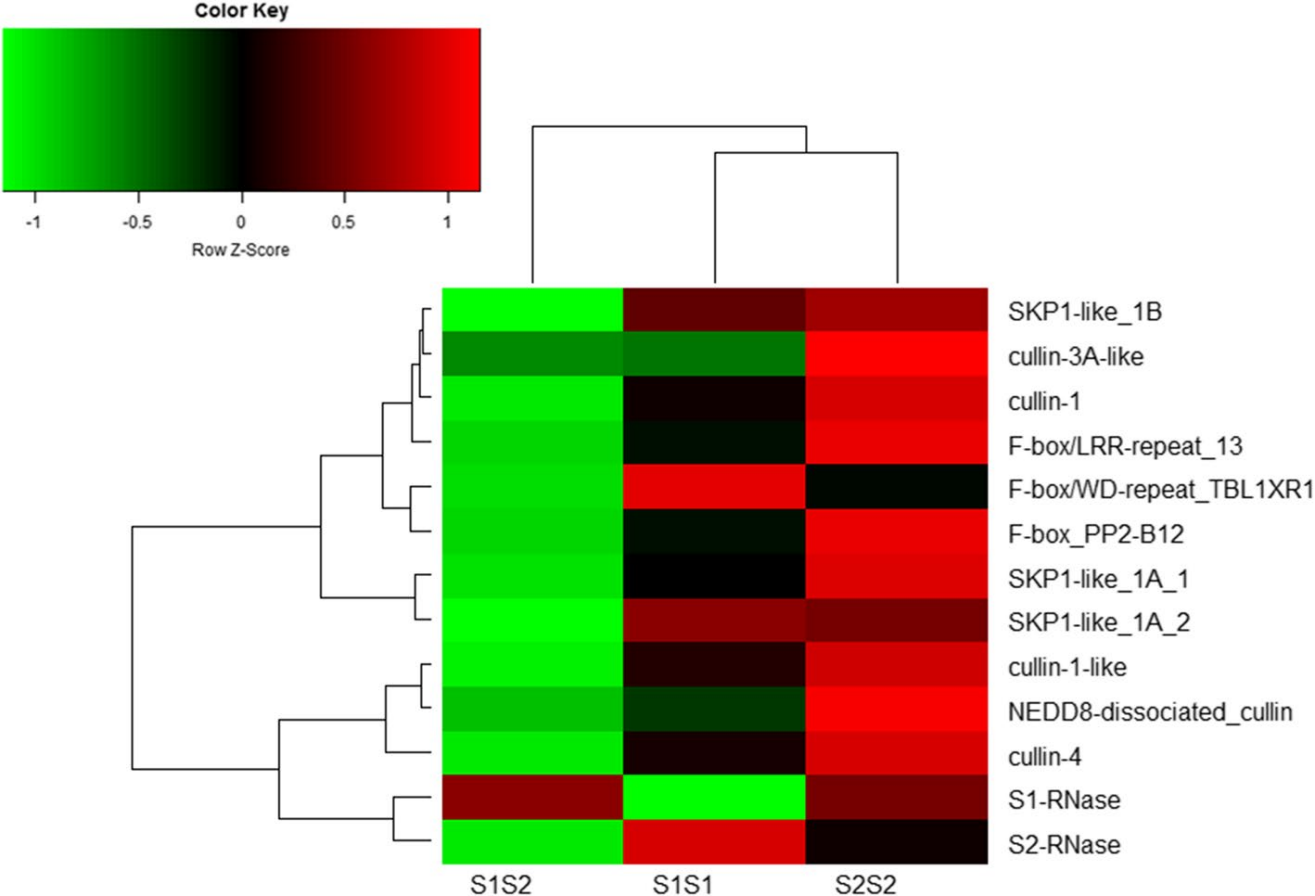
Heat-map showing expression levels of S-RNase-related proteins in styles of ‘S1S2’, ‘S1S1’, and ‘S2S2’

In this study, we conducted DEP analyses focusing on S-RNases and the proteins that interact with them in the styles of the three pear varieties. Figure 6 shows the heat-map of the expression levels of the 13 identified S-RNase-related proteins, which reflects their differences in expression levels across the three samples. In terms of the overall expression trend, using the offspring sample* S*_*1*_*S*_*1*_ as a normal expression standard, most of the S-RNase-related proteins were down-regulated in the parent S_*1*_S_*2*_ (Fig. 6). In contrast, in the offspring sample* S*_*2*_*S*_*2*_, most of the S-RNase-related proteins were up-regulated relative to their levels in the other offspring sample *S*_*1*_*S*_*1*_, with only a few exceptions. Compared with the parent *S*_*1*_*S*_*2*_, both offspring samples,* S*_*1*_*S*_*1*_ and *S*_*2*_*S*_*2*_, showed up-regulated expression of 12 out of the 13 S-RNase-related proteins, except for S_1_-RNase. Comparing *S*_*1*_*S*_*1*_ and *S*_*2*_*S*_*2*_, the expression levels of F-box/WD-repeat_TBL1XR1, SKP1-like_1A_2, and S_2_-RNase were higher in *S*_*1*_*S*_*1*_ than in *S*_*2*_*S*_*2*_, while the expression levels of the other 10 S-RNase-related proteins were higher in *S*_*2*_*S*_*2*_ than in* S*_*1*_*S*_*1*_*.*

## Discussion

Pollination for fertilization is the initial step in reproductive development, and it directly ensures the fruit setting rate. In flowering plants, SI serves as a ubiquitous mechanism to limit self-fertilization; it prevents pollen tubes that possess the same S-allele from growing normally in the pistil, so that they fail to reach the ovary for fertilization [43]. It is time-consuming and laborious to collocate trees of different pollen types and to conduct artificial pollination in production practice. Pollination failure in pear trees can also result from flowering periods that do not overlap and extreme weather, leading to serious economic losses. Therefore, research on the mechanism of SI in Rosaceae species is important from both theoretical and practical perspectives. In this study, we used a bud pollination method to create homozygous germplasm of *S*-alleles and verified their applicability in production. These are unique materials for further research on the SI mechanism and directed breeding.

### Self-pollination during the bud stage to overcome SI and creating homozygous single S-genotype germplasm

Artificial self-pollination at the bud stage can break down the obstacles of SI to achieve fruit setting. Selection of the optimum pollination period is crucial for the success of bud fertilization [44]. If pollination is carried out too early, the immature style contains insufficient nutrients for the heterotrophic pollen tubes to elongate, and the pollen tubes fail to weave through the style because of the compact tissue. If pollination is carried out too late, up-regulation of the S-gene protein results in recognition of self-pollen, triggering SI. The specific period for successful bud pollination has been explored in several species. Previous studies have shown that artificial self-pollination of tomato (*Solanum pennellii*) at 3–5 days before flowering can effectively avoid SI-related barriers. In tobacco (*N. tabacum*), pollination at late stage 7 (when the length of floral buds reaches 34–38 mm) resulted in 100% fruit set. Late stage 7 is accompanied by the development of anther primordia and the differentiation of specific anther tissue. In our study, we conducted self-pollination of ‘Huanghua’ pear at different bud periods (4, 6, and 8 days before flowering) and obtained the highest fruit set rate (43%) after pollination at 6 days before flowering.Wu et al. (2006) conducted a bud pollination experiment on pears, and the fruit setting rates of different varieties ranged from 18.2% to 82.1%. It can be seen that bud pollination can effectively overcome SI. But early pollination during the bud stage cannot complete self-fruitfulness, due to the style incomplete development [45]. The failure of the SI mechanism at the bud stage is associated with the absence (or only tiny amounts) of incompatible compounds in the immature pistil. These compounds include S-RNase in the Rosaceae and SRK in the Cruciferae. During style development in cabbage (*Brassica rapa* var. *pekinensis*), the abundance of SRK, a female determinant regulating SI, gradually increases from the budding to the maturity stage of the style, and the intensity of SI increases concurrently [46]. In Rosaceae species, silencing of the *S-RNase* gene created a self-fertile apple (*Malus* × *domestica* Borkh), with most of the unaffiliated pollen tubes reaching the base of the style to achieve fertilization. The average self-pollinated fruit setting rate increased from 4% in wild-type to 31% in the *S-RNase*-silenced line [47]. More specifically, the Chinese pear (*Pyrus bretschneideri*) cultivar ‘Yanzhuang’ lacks SI because of a mutation in the conserved glycine of the S-RNase C2 region [19].

We analyzed the spatio-temporal expression of S-RNase in the floral tissues of ‘Huanghua’ pear at different developmental stages (Fig. 1). The results show that the expression of S-RNase initiates at -4 DAF and gradually increases with style development, reaching the peak at 0 DAF. It is highly likely that it correlates with the formation of the SI mechanism, confirming once again that S-RNase serves as a female determinant of the SI mechanism in pear. Consequently, self-pollination at the bud stage is capable of breaking the limitations of SI to complete fertilization by circumventing the influence of the S-RNase. This method provides a reference for creating *S*-gene homozygotes in other GSI species.

### Some proteins are co-expressed with S-RNase in the style

**Fig. 7:**
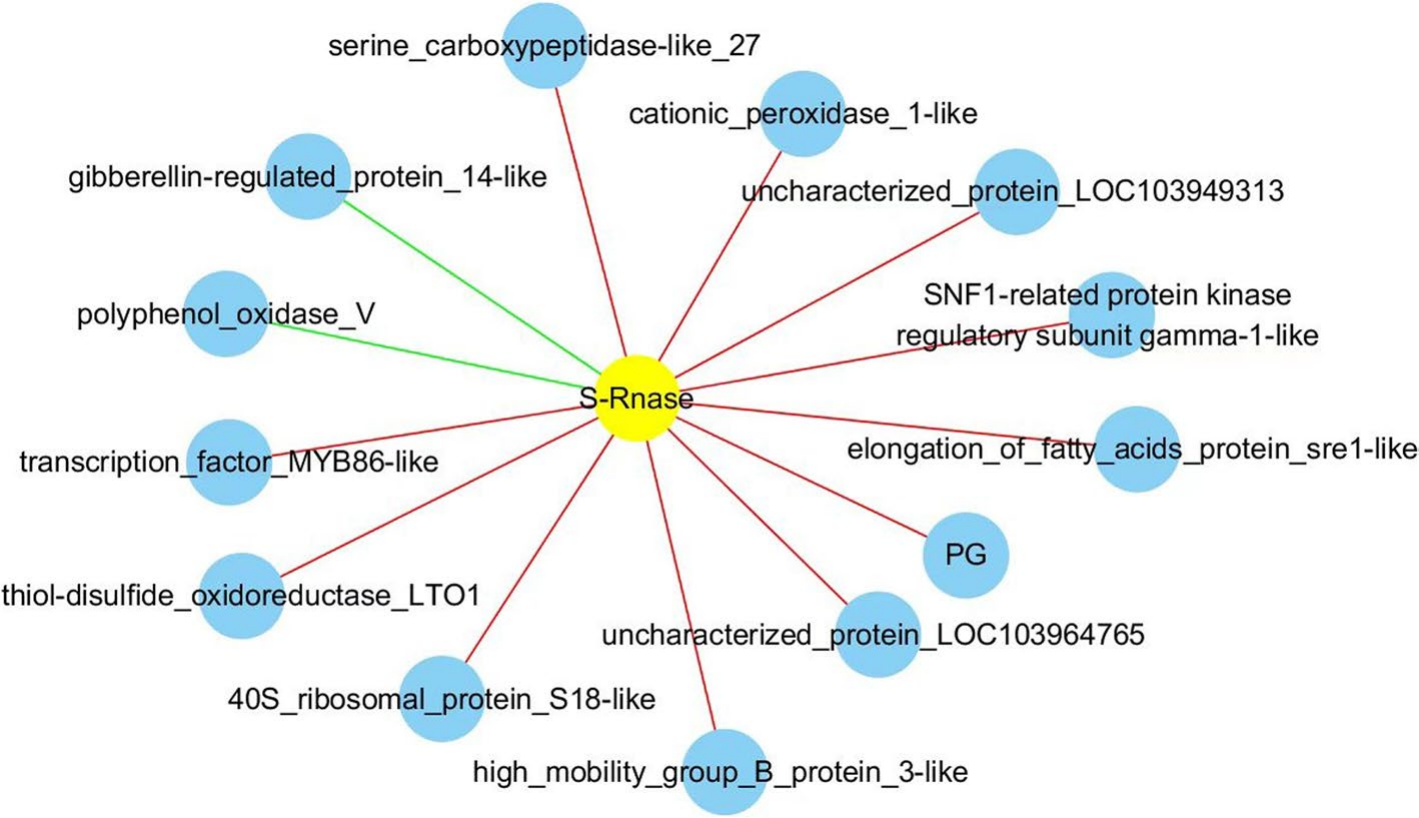
Co-expression network of S-RNase-related proteins in Pyrus styles

During the past decade, the transportation of S-RNase into the pollen tube, the signaling cascade of S-RNase toxicity have been studied. Three different pathways of pollen tube growth arrest by self S-RNase have been proposed [29]. The ABRE-binding factors may be involved in GSI by regulating the expression of genes related to pollen tube growth [28]. Leucine-rich repeat extensin (LRX) proteins are extracellular proteins that harbor an N-terminal leucine-richrepeat (LRR) domain and a C-terminal extensin domain [48]. Both PbLRXA2.1 and PbLRXA2.2 stimulated pollen tube growth and attenuated the inhibitory effects of self S-RNase on pollen tube growth by stabilizing the actin cytoskeleton and enhancing cell wall integrity [29]. A total of 13 S-RNase-related proteins were identified in the mass spectrometry analysis. The proteins could be divided into two categories: S-RNase family proteins, including S_1_-RNase and S_2_-RNase, and interactors of S-RNases, including skp1 family, cullin family, and F-box family proteins. These three types of proteins combine to form the SCF (Skp1-Cullins-F-box) complex, a multi-functional E3 ubiquitin ligase complex that can bind to S-RNase, leading to its oligo- or polyubiquitination. The complex is recognized by proteasomes and subsequently degraded, thus regulating the function of S-RNases (Fig. 7). Supplemental Tables 2 and 3 provide a summary of the changes in the expression levels of the 13 identified S-RNase-related proteins in the two sets of offspring samples relative to their parents, as well as the significance of the changes. As shown in the tables, the expression level of S_1_-RNase was significantly down-regulated in *S*_*1*_*S*_*1*_ compared with *S*_*1*_*S*_*2*_, while that of S_2_-RNase was not significantly different among the three samples. The expression level of the S-RNase-related protein F-box/WD-repeat_TBL1XR1 was significantly up-regulated in the *S*_*1*_*S*_*1*_ offspring relative to the parent *S*_*1*_*S*_*2*_, but it was not significantly different between the *S*_*2*_*S*_*2*_ offspring and the parent *S*_*1*_*S*_*2*_. The expression levels of F-box_PP2-B12 and F-box/LRR-repeat_13 were significantly up-regulated in the offspring sample* S*_*2*_*S*_*2*_ compared with the parent* S*_*1*_*S*_*2*_, but there was no significant difference in their expression levels in the *S*_*1*_*S*_*1*_ offspring sample compared with the parent *S*_*1*_*S*_*2*_. This may indicate that the regulatory pathways involving S-RNase include different sets of associated proteins in the two offspring samples, *S*_*1*_*S*_*1*_ and* S*_*2*_*S*_*2*_. These results provide a reference for further research on S-RNases and their related interacting proteins in styles.

### Homozygous S-gene germplasms are excellent materials for further research on SI mechanism and pear industry

Different S-allele genotypes are classified according to significant differences in the location of the SFBB/F-box genes relative to the S-RNase gene. However, this complex diploid genotype is not conducive to investigating the differences between pollen-specific recognition and pistil S-glycoprotein-specific recognition. In previous studies, the isolation of pure single S-gene proteins has been achieved by means of heterologous expression. For example, expression of the *PbrS-RNase* recombinant vector in *Escherichia coli* provided sufficient protein to perform biochemical analyses in vitro, thereby facilitating exploration of the detailed SI mechanism. That study revealed a new pathway in which PbrS-RNase induces the death of incompatible pollen tubes by directly binding to, and cleaving, F-actin [30]. However, single *S*-gene purified proteins are restricted to in vitro experimental studies and are not suitable for industrial applications in fruit trees. In this study, we obtained two S-RNase homozygous germplasm materials (*S*_*1*_*S*_*1*_ and *S*_*2*_*S*_*2*_) using the bud self-pollination method. This makes extracting single S-RNase from pistils more convenient. It also can verify relevant SI mechanisms in vivo. These are excellent materials for further research on the SI mechanism from both theoretical and practical perspectives.

During SI pollination, pollen grains germinate into the style and the S-RNase is internalized into the pollen tube for recognition responses [49]. The same type of S-RNase will not be eliminated by the ubiquitination system and will eventually be released to exercise its cytotoxic effects, thereby inducing the typical SI response. This response includes depolymerization of microfilament structures, increased levels of reactive oxygen species, and PCD in unaffiliated pollen tubes. In our study, self-pollination of ‘*S*_*1*_*S*_*2*_’, ‘*S*_*1*_*S*_*1*_’, and ‘*S*_*2*_*S*_*2*_’ resulted in typical SI responses. Notably, the homozygous germplasms ‘*S*_*1*_*S*_*1*_’ and ‘*S*_*2*_*S*_*2*_’ were cross-compatible when used as the female parent, but cross-incompatible when used as the male parent. This implies that it will be useful to develop homozygous germplasms as major cultivars for pear orchard production.

In practice, pear trees must be pollinated with pollen with different S-genotypes as compared to the main cultivar to ensure a good fruit setting rate. At present, the main cultivars in China include ‘Dangshansu’ (*S*_*7*_*S*_*34*_), ‘Cuiguan’ (*S*_*3*_*S*_*5*_), ‘Huangguan’ (*S*_*4*_*S*_*16*_), and ‘Zhongli No. 1’ (*S*_*4*_*S*_*35*_), none of which contain *S*_*1*_ or *S*_*2*_. It is reasonable to match homozygous germplasm (*S*_*1*_*S*_*1*_ and *S*_*2*_*S*_*2*_) to completely circumvent the limitations of SI, thereby further safeguarding the fruit setting rate. In addition, these homozygous germplasm materials can serve as stable donors for efficiently integrating *S*_*1*_ and *S*_*2*_ into the genome of the main cultivar. New varieties can be developed with multiple functions as both the main cultivar and as an effective pollinator.

## Conclusion

This study describes a reliable method for creating homozygous single *S*-genotype germplasm, which will accelerate fruit tree breeding programs by allowing for the directional insertion of *S*-alleles. This will be useful for the continuous enrichment of pear pollinator resources with different genotypes to promote the development of the pear industry.

### Supplementary Information

**Additional file 1.** Suplemental data.

## Data Availability

The mass spectrometry proteomics data have been deposited to the ProteomeXchange Consortium via the PRIDE partner repository with the dataset identifier PXD043543.
