# Supplementary material for: Specific functions of single pistil S-RNases in S-gene homozygous Pyrus germplasm
Source: BMC Plant Biol. 2023 Nov 20;23:578. doi: 10.1186/s12870-023-04605-0 (PMC10658986; doi:10.1186/s12870-023-04605-0)
Supplement: Supplementary file 1 — Additional file 1. Suplemental data. [file 12870_2023_4605_MOESM1_ESM.ppt]

## Slide 1
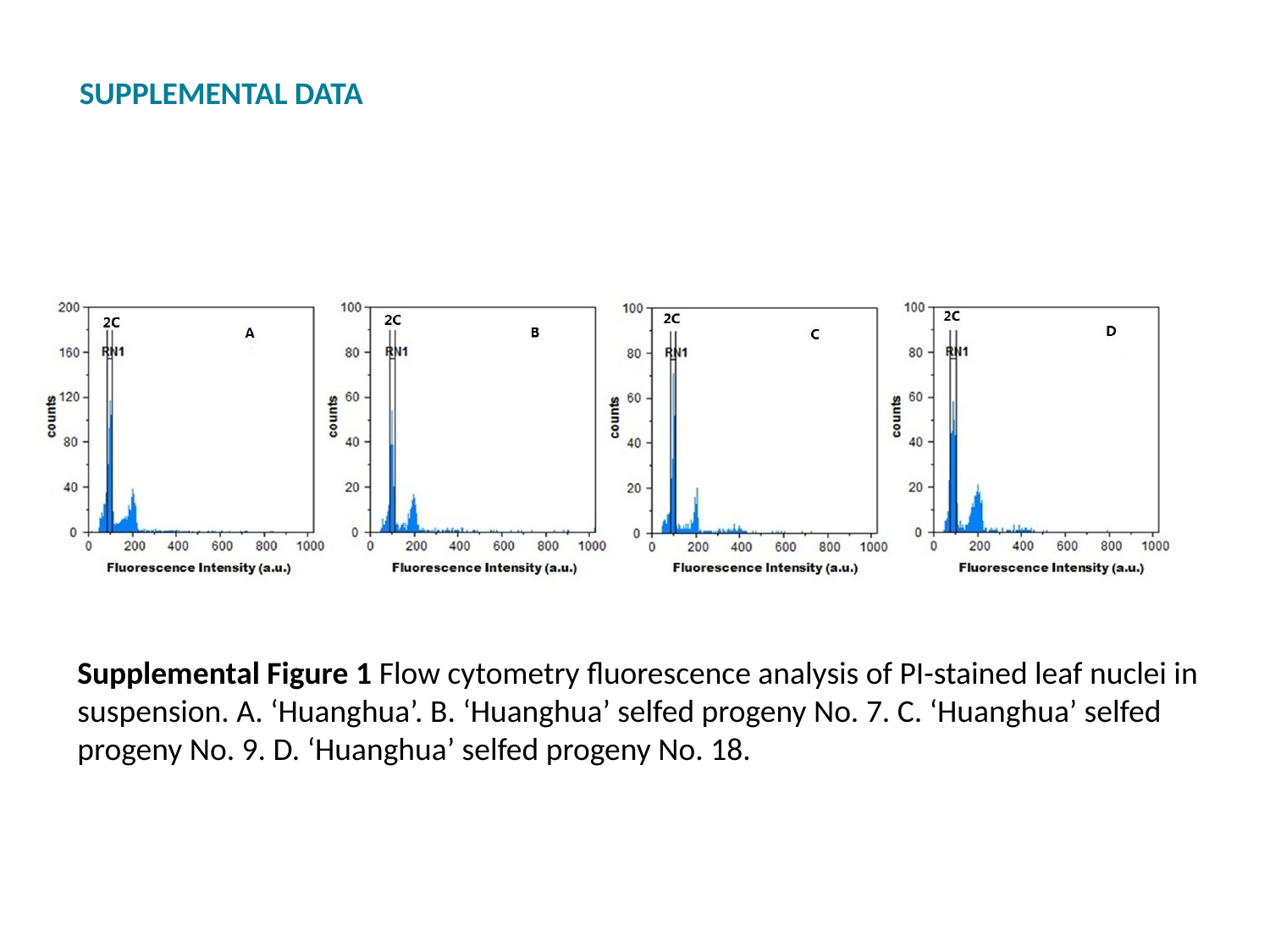

SUPPLEMENTAL DATA
Supplemental Figure 1 Flow cytometry fluorescence analysis of PI-stained leaf nuclei in suspension. A. ‘Huanghua’. B. ‘Huanghua’ selfed progeny No. 7. C. ‘Huanghua’ selfed progeny No. 9. D. ‘Huanghua’ selfed progeny No. 18.

## Slide 2
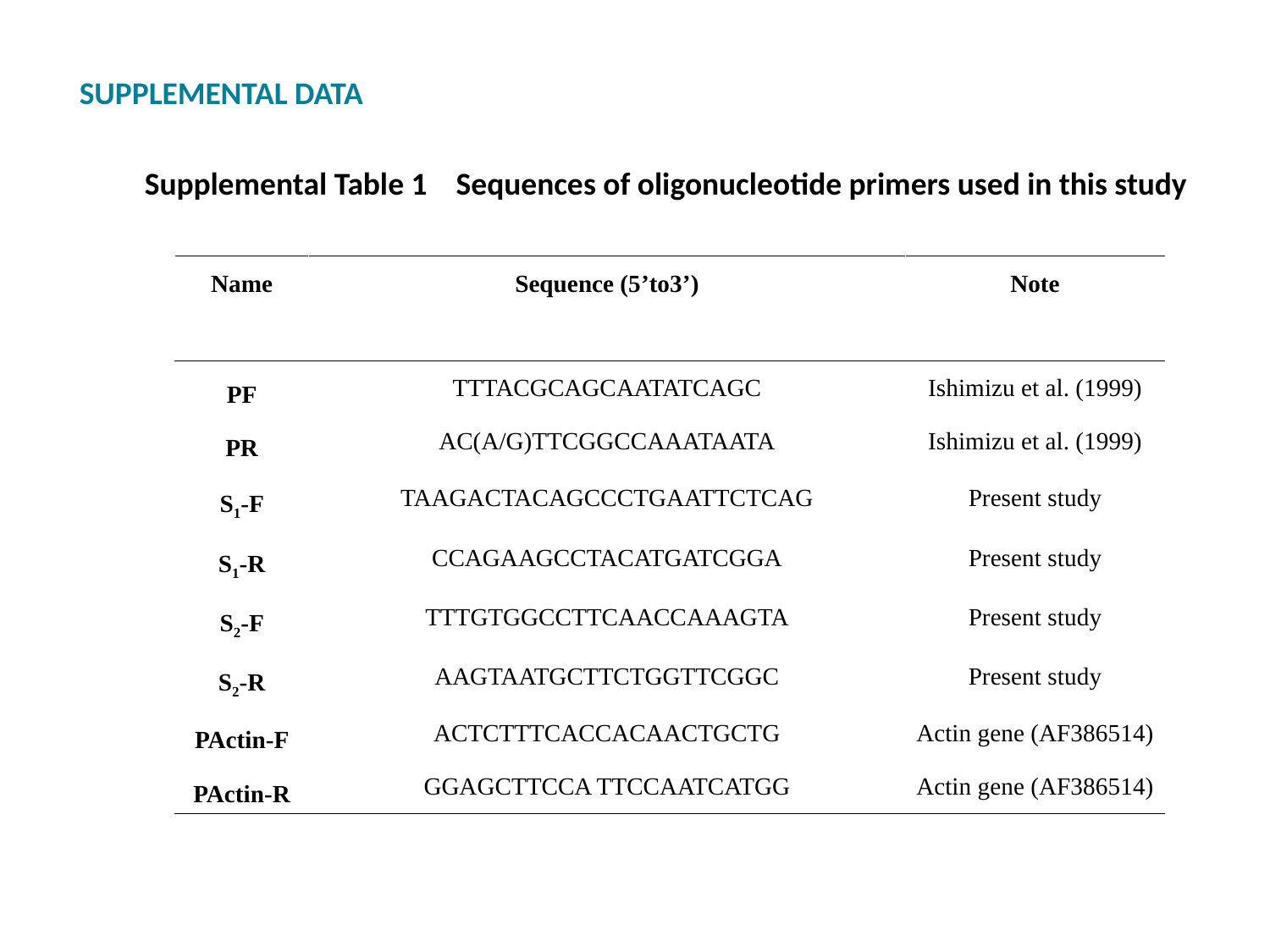

SUPPLEMENTAL DATA
Supplemental Table 1 Sequences of oligonucleotide primers used in this study
| Name | Sequence (5’to3’) | Note |
| --- | --- | --- |
| PF | TTTACGCAGCAATATCAGC | Ishimizu et al. (1999) |
| PR | AC(A/G)TTCGGCCAAATAATA | Ishimizu et al. (1999) |
| S1-F | TAAGACTACAGCCCTGAATTCTCAG | Present study |
| S1-R | CCAGAAGCCTACATGATCGGA | Present study |
| S2-F | TTTGTGGCCTTCAACCAAAGTA | Present study |
| S2-R | AAGTAATGCTTCTGGTTCGGC | Present study |
| PActin-F | ACTCTTTCACCACAACTGCTG | Actin gene (AF386514) |
| PActin-R | GGAGCTTCCA TTCCAATCATGG | Actin gene (AF386514) |

## Slide 3
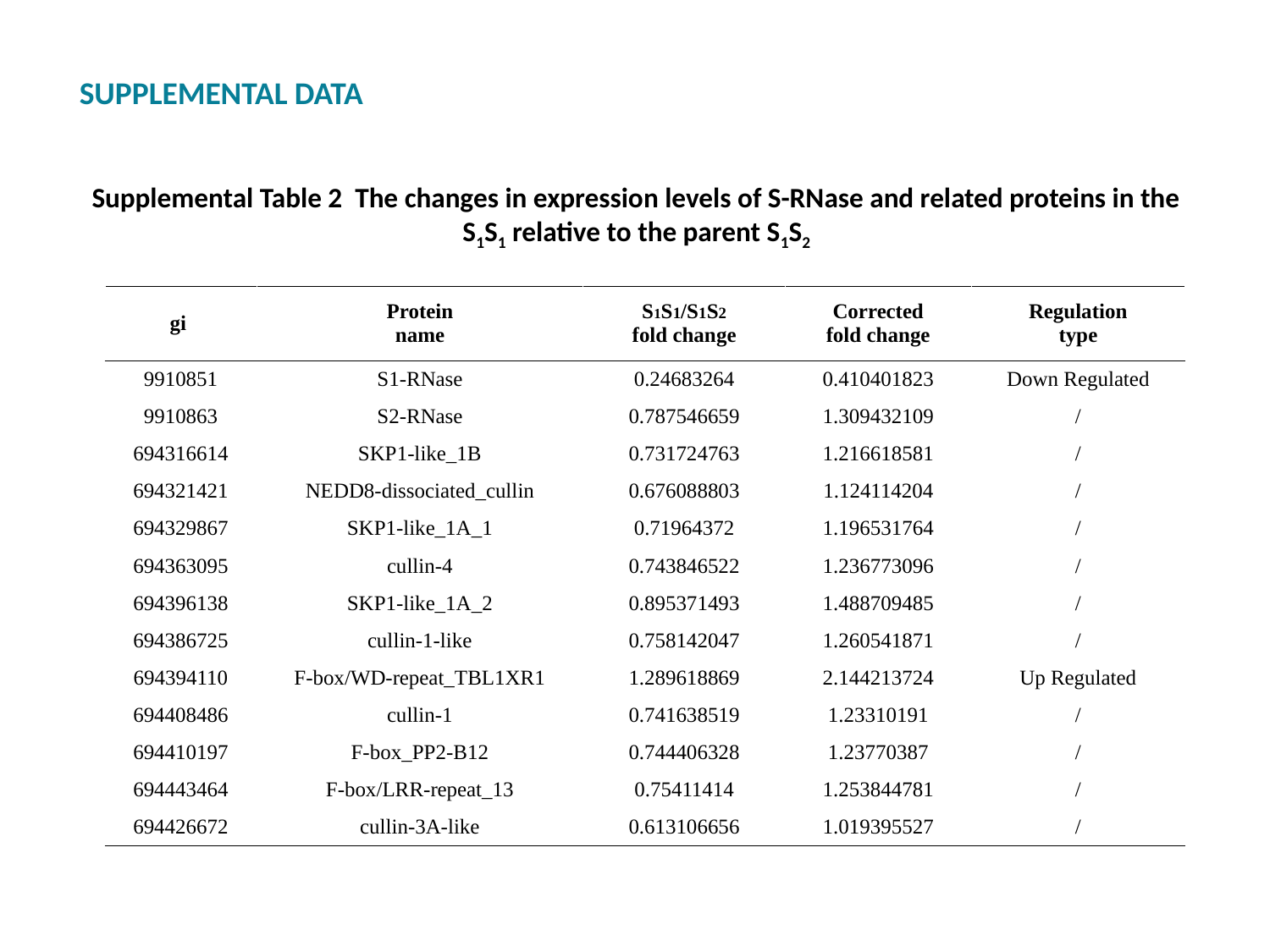

SUPPLEMENTAL DATA
# Supplemental Table 2 The changes in expression levels of S-RNase and related proteins in the S1S1 relative to the parent S1S2
| gi | Protein name | S1S1/S1S2 fold change | Corrected fold change | Regulation type |
| --- | --- | --- | --- | --- |
| 9910851 | S1-RNase | 0.24683264 | 0.410401823 | Down Regulated |
| 9910863 | S2-RNase | 0.787546659 | 1.309432109 | / |
| 694316614 | SKP1-like\_1B | 0.731724763 | 1.216618581 | / |
| 694321421 | NEDD8-dissociated\_cullin | 0.676088803 | 1.124114204 | / |
| 694329867 | SKP1-like\_1A\_1 | 0.71964372 | 1.196531764 | / |
| 694363095 | cullin-4 | 0.743846522 | 1.236773096 | / |
| 694396138 | SKP1-like\_1A\_2 | 0.895371493 | 1.488709485 | / |
| 694386725 | cullin-1-like | 0.758142047 | 1.260541871 | / |
| 694394110 | F-box/WD-repeat\_TBL1XR1 | 1.289618869 | 2.144213724 | Up Regulated |
| 694408486 | cullin-1 | 0.741638519 | 1.23310191 | / |
| 694410197 | F-box\_PP2-B12 | 0.744406328 | 1.23770387 | / |
| 694443464 | F-box/LRR-repeat\_13 | 0.75411414 | 1.253844781 | / |
| 694426672 | cullin-3A-like | 0.613106656 | 1.019395527 | / |

## Slide 4
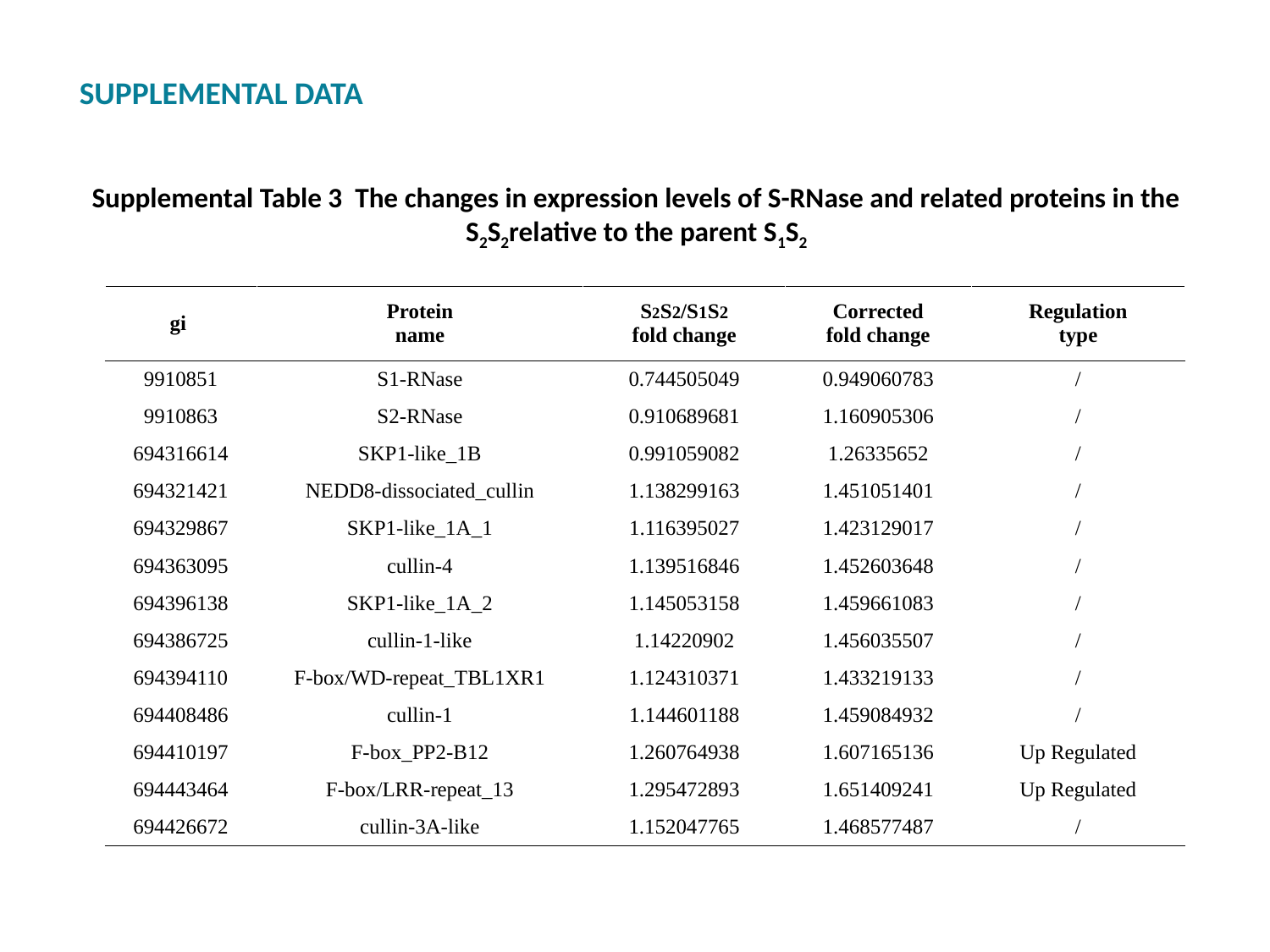

SUPPLEMENTAL DATA
# Supplemental Table 3 The changes in expression levels of S-RNase and related proteins in the S2S2relative to the parent S1S2
| gi | Protein name | S2S2/S1S2 fold change | Corrected fold change | Regulation type |
| --- | --- | --- | --- | --- |
| 9910851 | S1-RNase | 0.744505049 | 0.949060783 | / |
| 9910863 | S2-RNase | 0.910689681 | 1.160905306 | / |
| 694316614 | SKP1-like\_1B | 0.991059082 | 1.26335652 | / |
| 694321421 | NEDD8-dissociated\_cullin | 1.138299163 | 1.451051401 | / |
| 694329867 | SKP1-like\_1A\_1 | 1.116395027 | 1.423129017 | / |
| 694363095 | cullin-4 | 1.139516846 | 1.452603648 | / |
| 694396138 | SKP1-like\_1A\_2 | 1.145053158 | 1.459661083 | / |
| 694386725 | cullin-1-like | 1.14220902 | 1.456035507 | / |
| 694394110 | F-box/WD-repeat\_TBL1XR1 | 1.124310371 | 1.433219133 | / |
| 694408486 | cullin-1 | 1.144601188 | 1.459084932 | / |
| 694410197 | F-box\_PP2-B12 | 1.260764938 | 1.607165136 | Up Regulated |
| 694443464 | F-box/LRR-repeat\_13 | 1.295472893 | 1.651409241 | Up Regulated |
| 694426672 | cullin-3A-like | 1.152047765 | 1.468577487 | / |
